# Extending the “One Strain Many Compounds” (OSMAC) Principle to Marine Microorganisms

**DOI:** 10.3390/md16070244

**Published:** 2018-07-23

**Authors:** Stefano Romano, Stephen A. Jackson, Sloane Patry, Alan D. W. Dobson

**Affiliations:** 1 Division of Microbial Ecology, Department of Microbiology and Ecosystem Science, Althanstraße 14, 1090 Vienna, Austria; 2 School of Microbiology, University College Cork, National University of Ireland, T12 YN60 Cork, Ireland; sjackson@ucc.ie (S.A.J.); sloane.patry@gmail.com (S.P.); 3 Environmental Research Institute, University College Cork, National University of Ireland, Lee Road, T23 XE10 Cork, Ireland

**Keywords:** secondary metabolites, biosynthetic gene clusters, cultivation, environmental cues, co-cultivation, chemical elicitors, one strain many compounds, OSMAC

## Abstract

Genomic data often highlights an inconsistency between the number of gene clusters identified using bioinformatic approaches as potentially producing secondary metabolites and the actual number of chemically characterized secondary metabolites produced by any given microorganism. Such gene clusters are generally considered as “silent”, meaning that they are not expressed under laboratory conditions. Triggering expression of these “silent” clusters could result in unlocking the chemical diversity they control, allowing the discovery of novel molecules of both medical and biotechnological interest. Therefore, both genetic and cultivation-based techniques have been developed aimed at stimulating expression of these “silent” genes. The principles behind the cultivation based approaches have been conceptualized in the “one strain many compounds” (OSMAC) framework, which underlines how a single strain can produce different molecules when grown under different environmental conditions. Parameters such as, nutrient content, temperature, and rate of aeration can be easily changed, altering the global physiology of a microbial strain and in turn significantly affecting its secondary metabolism. As a direct extension of such approaches, co-cultivation strategies and the addition of chemical elicitors have also been used as cues to activate “silent” clusters. In this review, we aim to provide a focused and comprehensive overview of these strategies as they pertain to marine microbes. Moreover, we underline how changes in some parameters which have provided important results in terrestrial microbes, but which have rarely been considered in marine microorganisms, may represent additional strategies to awaken “silent” gene clusters in marine microbes. Unfortunately, the empirical nature of the OSMAC approach forces scientists to perform extensive laboratory experiments. Nevertheless, we believe that some computation and experimental based techniques which are used in other disciplines, and which we discuss; could be effectively employed to help streamline the OSMAC based approaches. We believe that natural products discovery in marine microorganisms would be greatly aided through the integration of basic microbiological approaches, computational methods, and technological innovations, thereby helping unearth much of the as yet untapped potential of these microorganisms.

## 1. Introduction

Natural products (NPs) are organic molecules produced by living organisms, and in the last century, their applications have underpinned fundamental advances in both the industrial and medical fields. Between 50% and 70% of today’s small molecule-based therapeutics have originated from NPs [1], underlying the pivotal role these compounds play in modern medicine. The majority of medically relevant NPs are secondary metabolites (SMs), which are generally and historically defined as molecules not essential for the survival of the producing organism but which aid in increasing its competitiveness. More than 10% of bioactive NPs are microbial in origin, and in the last decades, microorganisms have assumed a central role in the reinvigorated field of NP discovery [2]. Microbial NPs have proven useful in the treatment of viral, fungal, and bacterial infections, as well as in the treatment of various cancers. However, by their very nature pathogenic microbes and cancer cells are evolving entities, which are increasingly developing resistance mechanisms against the latest generation of medical treatments. Such phenomena have become some of the most pressing issues in modern society. In particular, antimicrobial resistance (AMR) has been recognized as one of the main threats to humanity, with the frightening prediction of ~10 million deaths due to AMR by 2050, if immediate action is not taken [3]. Although on average ~1000 new NPs have been discovered each year over the last two decades, the recurrent rediscovery of already known compounds continues to be a major bottleneck in the field [4]. Recent analyses have however underlined that appreciable numbers of NPs with novel chemical features or scaffolds are still being discovered every year, even though the frequency of such findings has been decreasing over time [4]. This scenario has encouraged scientists to explore less accessible and until now less investigated environments and ecosystems such as those found in the oceans, and in fact marine natural products are assuming an increasingly central role in the search for novel bioactivities. These environments cover >70% of the earth’s surface, and contain 95% of the biosphere, including millions of phylogenetically divergent microorganisms, thus offering an as yet largely untapped treasure of chemical biodiversity [5,6]. Moreover, marine environments have peculiar geochemical features when compared with other land-based ecosystems, which is often reflected in the chemistry and properties of marine NPs [7].

The advent of high throughput DNA sequencing technologies has contributed to reinvigorating the interest in microbial NPs, with the number of sequenced microbial genomes exploding over the last few years. At the time of writing this review, the IMG database contained 61350 bacterial, 1441 archaeal, and 698 eukaryotic genome sequences. This “revolution” in genomic information has highlighted an inconsistency between the number of gene clusters that based on bioinformatics analyses could potentially be involved in secondary metabolite production and the actual total number of chemically characterized SMs for a given organism. In general, SMs are produced by enzymes encoded by co-localized genes, forming so-called biosynthetic gene clusters (BGCs). Genome sequencing has revealed that microbes often harbor various “cryptic” BGCs, which are not associated with chemically characterized molecules. These BGCs are thought to be “silent” (not expressed) under laboratory conditions. For example, the model actinomycete *Streptomyces coelicolor* was initially known to encode BGCs for the production of 13 different classes of SMs, but in 2002 analysis of the newly sequenced genome revealed the presence of 16 additional BGCs which likely produce molecules with novel structures [8]. The production of SMs is not cost free for the cell. Both energy and resources need to be invested in the production of the biosynthetic enzymes, the molecular precursors, together with the assembly, maturation, and tailoring of the final compound. Therefore, the synthesis of SMs is tightly regulated and believed to be activated in response to specific environmental conditions; potentially helping to increase the fitness of the producing organisms [9,10]. This is believed to be the main reason why many BGCs are actually “silent” when the producing strains are cultured under standard laboratory conditions. However, the lack of appropriate bioassays to detect bioactivities associated with unknown molecules could also play a role in the underestimation of microbial NPs. On the other hand, under laboratory conditions some of the “silent” BGCs may in fact be expressed but at very low levels, resulting in metabolite titers which are below analytical detection. In such cases, even bioassay-guided fractionation approaches may not help in identifying these low abundance molecules, as the more abundantly produced SMs would mask the bioactivity of these compounds. In fact, the genetic “knock-down” of genes encoding known BGCs can help in the detection of less abundantly produced molecules [11].

It is widely believed that thus far we have only considered “the tip of the microbial NP iceberg”. This together with the realization that microbial diversity within marine ecosystems is even greater than previously imagined, has resulted in the development of both cultivation-dependent and independent methods to help unlock the potential of these “silent” BGCs. Such approaches can be broadly divided into methods that use genetic engineering techniques to homologously or heterologously express the BGCs, and methods that are based on modifying the cultivation conditions in which the producing organism is grown; with the intention of providing an appropriate environment to trigger the expression of the “silent” BGCs. Methods that generate global physiological changes and methods that are pathway-specific belong to the first group of approaches [12]. The selection of strains having mutations in RNA polymerase or ribosomal proteins, as well as either deletion or induction of global transcription regulators, are all techniques that have been successfully employed in both terrestrial and marine microorganisms to alter gene regulation in producing strains, thereby inducing expression of “silent” BGCs [12,13]. Similarly, in eukaryotic microorganisms, genetic manipulation to alter epigenetic processes, which are responsible for covalent modification of DNA and chromatin, have led to global changes in transcription profiles and to SM production [12,13]. Conversely, pathway-specific strategies allow a more precise control of the homologous or heterologous expression of BGCs. For example, these methods rely on the expression of pathway-specific activator genes or deletion of pathway-specific repressor genes. Similarly, strong promotors can be added to the BGC of interest to stimulate expression within the original producing organism. Alternatively, pathways can be cloned into heterologous hosts, which have easily controllable transcriptional machineries [12,13]. All these approaches relying on genetic manipulations have so far resulted in invaluable advances in the field, leading to the discovery of novel bioactive NPs. Moreover, such approaches are of particular interest in the creation of “synthetic” pathways, via genetically engineering new versions of BGCs by modifying the gene arrangements of known and well-characterized pathways [13,14].

Nevertheless, these methods require a relatively high degree of knowledge of the biology of the producing organisms, including knowledge of both the overall transcription regulatory networks and the features and regulation of the encoded BGCs. In the first instance, a BGC of interest needs to be identified. Although new bioinformatic based approaches provide considerable help in the identification of BGCs and can help in predicting the tentative structure of the SM which is likely to be produced [15], these analyses are not always completely accurate and may not always allow the successful identification of lesser known BGCs. For example, the gene cluster for production of the potent antibiotic tropodiethetic acid (TDA) cannot be easily predicted by the majority of currently available bioinformatic tools. Moreover, in order to heterologously or homologously manipulate expression of the BGCs, insights are needed into the minimal set of genes involved in the biosynthetic process. In many instances this is not a trivial task as, for example, BGC boundaries are not always straightforward to identify [16]. Also, there are many examples of “talented” microorganisms, from a NP production standpoint; especially from the marine environment; which are not easy to manipulate genetically, limiting most of the aforementioned genetic engineering approaches to the better known and more studied phylogenetic groups [12,13,17,18]. Finally, it is also important to point out that the production of SMs can require tight regulation of central physiological features and the coordinated expression of different pathways. In fact, it has been shown that multiple compounds can derive from a single pathway [19,20,21,22], with environmental factors and intracellular precursor abundances influencing the type of metabolite produced [19]. Moreover, multiple BGCs can be involved in the synthesis of a single SM. For example, in *Rhodococcus jostii* synthesis of the siderophore rhodochelin has been shown to require the coordinated expression of three independent BGCs, located in different chromosomal regions [23]. There are also cases where molecules produced by a pathway are modified by a second pathway whose expression is then triggered by the modified molecule [24]. Similarly, even in the well-studied *Streptomyces* genus, which are prolific producers of NPs, novel synthetic routes relying on the reciprocal dependence of two BGCs have been recently discovered, resulting in three different pyrrolamides being produced by two BGCs [25]. If information such as this is not previously known, such type of metabolic interaction cannot be accounted for during the pathway-specific genetic manipulations. Therefore, in such cases, the global alteration of microbial physiology can be more useful in changing the SM profile of a strain. 

**Figure 1:**
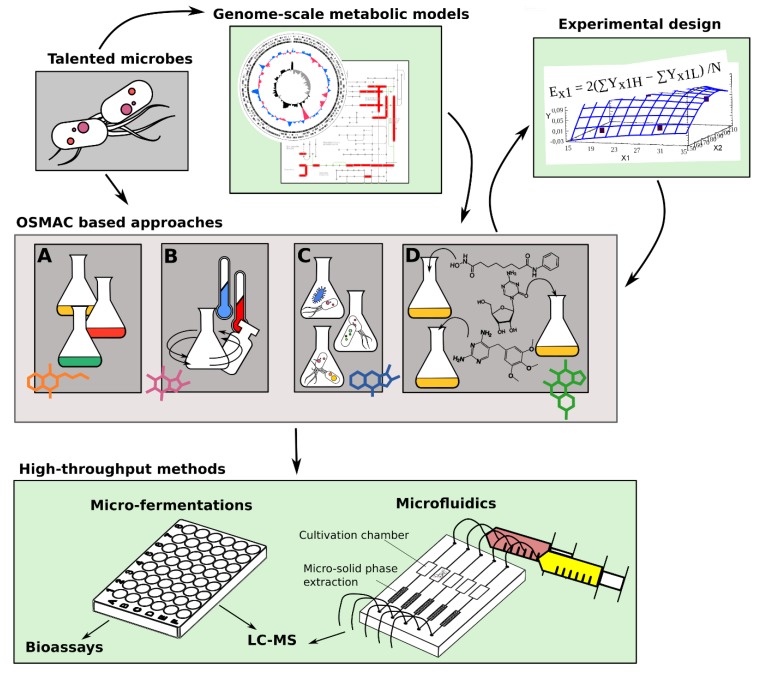
Overview of the “one strain many compounds” (OSMAC) based approaches to elicit production of cryptic SMs. In the green boxes are reported the strategies we propose as tools to streamline the experimental procedures underpinning the OSMAC approaches. Full description of the latter is reported in the sections of the review: “Nutrient regimes” (**A**); “Physical parameters” (**B**); “Co-cultivation” (**C**); “Chemical elicitors” (**D**). The micro-fermentations work-flow was inspired by the work of Adnani et al. [27], whereas the microfluidic device was inspired by the examples reported in the review of Jie et al. [28].

Similar global physiological alterations are triggered by all those approaches based on the modification of the growth conditions without genetically manipulating the producing organism. These approaches derive from fermentation optimization practices, which were historically used in industrial microbiology to ameliorate biotechnological processes. The general framework was originally conceptualized by Zeeck and colleagues, who postulated the “one strain many compounds” (OSMAC) approach [26]. The basic idea behind this approach is that each microbial strain has the potential to produce multiple compounds, but only subsets of these compounds are produced under specific growth conditions. Therefore, variations in cultivation parameters can elicit the production and discovery of new SMs. By changing cultivation parameters such as temperature, salinity, aeration, and even the shape of the flasks, Zeeck and colleagues demonstrated that the fungus *Aspergillus ochraceus*, which was thought to only produce the metabolite aspinonene, was able to produce 15 additional metabolites [26]. Subsequently, studies conducted in the past 20 years, have revealed a plethora of cultivation parameters that if modified affect SM production in microorganisms, including various nutrients, trace elements, physical parameters (i.e., pH, temperature), and addition of chemical elicitors (i.e., sub-lethal concentrations of antibiotics, communication molecules). Moreover, co-cultivation of microbes, and addition of factors affecting epigenetic control can also be framed within the OSMAC principle. All of these changes to the cultivation processes offer a broad arsenal of strategies which all rely on altering the cellular machinery of the producing organism in response to various growth conditions. The main advantage of these approaches is that they do not require an *a*-*priory* knowledge of the type of BGCs and/or regulatory processes governing their expression. Moreover, these approaches can be applied to microbes which are less amenable to genetic manipulation, and represent ideal tools which can in particular be employed to unlock genetically refractory marine microorganisms [13,18]. On the other hand, such approaches can be both time-consuming and challenging from a practical standpoint, as the number of potential changes in the cultivation parameters can be enormous. Here, we report on the more promising cultivation parameters affecting SM production in marine microorganisms (Figure 1). Notwithstanding the numerous studies aimed to elicit activation of “silent” BGCs using the OSMAC principle, much remains to be discovered, particularly for marine microorganisms. In order to stimulate interest in these approaches, we additionally provide a summary of new technologies which we feel could help to streamline the cultivation procedures, thus increasing overall throughput in the NP discovery pipeline (Figure 1).

## 2. Change in Nutrient Regimes

A textbook example of the effects of nutrient availability on microbial SM production is the link between environmental iron concentrations and siderophore production. The latter are SMs released by microbes to scavenge iron, when the iron concentration is limiting in the environment. However, the effects of nutrient concentration on the production of SMs are much more complex and vary quite markedly between different microorganisms. One of the earliest examples of the effect of nutrients on the production of secondary metabolites by marine microbes was the report on an antibiotic produced by the marine actinomycete *Chainia purpurogena*, where production was dependent on the addition of an extract from the seaweed *Laminaria* [29]. Some of these effects, for example that of different carbon sources and of phosphate limitation, have been the subject of ongoing studies; and rely on complex regulatory processes that are often interconnected to regulatory circuits affecting overall microbial cellular physiology [30,31]. More recently trace elements have also been reported to have a marked effect on SM production; with a number of studies considering these effects in both eukaryotic and prokaryotic marine microorganisms, leading to the discovery of a variety of novel compounds [32,33]. 

### 2.1. Carbon Source

A consideration of the types of carbon sources available to a microorganism and their effects on secondary metabolism is a logical starting point when taking account of the potential effects of different nutrient regimes. Carbon catabolite repression is a well-known phenomenon with respect to exerting global control over gene expression in the microbial world, and has been exhaustively reviewed by Sanchez and co-workers [30]. In addition, Martin and colleagues have also described the global regulatory control of transcription in *Streptomyces coelicolor*, involving the effects of both single and combined nutrient sources; including the effects of the sources and relative concentrations of carbon and phosphorous or carbon and nitrogen [31]. These valuable reviews provide a good starting point for the design of different growth media to investigate SM production in marine microorganisms. Various studies have reported the effect of carbon sources on both the typology and titration of the SMs produced. For example, the variable effects of different carbon sources on the production of anti-tumor glycolipids, produced by the marine-derived fungus *Acremonium* sp. MMS540, was the subject of an investigation where six different culture conditions, including the use of glucose or galactose as carbon sources was investigated [34]. Both total lipid and lipid class compositions were assessed together with glycolipid modifications induced by sugar source changes. While the replacement of glucose with galactose did not result in any dramatic alteration in total lipid production, a major modification in the ratio of the main fatty acids produced with a concomitant decrease in the diversity of the minor fatty acid profile was observed [34]. 

The Gram group has reported stimulation of the production of the antibiotic andrimid by chitin in the marine bacterium *Vibrio coralliiltycus* S2052; which is a pathogen of corals and crustaceans [35]. Chitin is the most abundant renewable biopolymer in the oceans and is thus a ubiquitous carbon source in marine environments. Andrimid production was more than double in media containing chitin as the sole carbon source relative to glucose grown cells. In further experiments the ability of *V. coralliilyticus* to use other abundant marine carbon sources was tested. Differential utilization was observed when the strain was grown on live shrimp as a natural chitin source, and on aqueous algal extracts of *Fucus* sp. and *Laminaria* sp. macroalgae; with *V. coralliilyticus* utilizing alginate but not fucoidan or laminarin. Interestingly, while growing on alginate no andrimid production was observed. Further work was undertaken by the group employing genome mining, transcriptomics and metabolomics-based approaches to further investigate andrimid production in *V. coralliilyticus* [36]. They reported that five BGCs in the vibrio genome were differentially expressed when grown on chitin rather than on glucose, with andrimid production again being higher in cultures grown on chitin than on glucose. The authors suggested an ecological model whereby *V. coralliilyticus* produces andrimid during colonization of natural marine chitin sources such as when infecting shrimp, which further illustrates the complex niche adaptive regulation of SM production. This finding built on previous work by the same group where they reported that genes involved in secondary metabolism in *Vibrio coralliilyticus* S2052 and *Photobacterium galatheae* S2753 were up-regulated by growth on chitin, with andrimid and holomycin production being higher when the strains were grown using this polymer [37].

Fourteen different single carbon sources were assessed to determine optimal growth conditions for antibiotic production of the antibiotic SBR-22 in *Streptomyces psammoticus* BT-408, an isolate from marine sediments which effectively inhibited the growth of MRSA (methicillin resistant *Staphylococcus aureus*) [38]. Large variations in the yield of SBR-22 were observed, ranging from 20 µg/mL when *S. psammoticus* was grown on arabinose to 190 µg/mL when grown on glucose. Similarly, glucose was also the substrate that allowed maximal antibiotic production in another marine actinobacterium strain [39]. This isolate, which is taxonomically related to streptomycetes but which was not further characterized, displayed broad range antimicrobial activities with no evidence of carbon catabolite repression. Similarly, no carbon catabolite repression was observed in a marine sediment-derived *Pseudoalteromonas piscicida* strain, which was the object of a study that investigated the effect of nitrogen source, pH, NaCl concentration, incubation temperature and incubation time, as well as carbon source on anti-MRSA antibiotic production. In this strain optimal antibiotic production was also observed when the strain was grown on glucose [40]. Conversely, in the aquaculture probiotic strain *Pseudomonas* sp. MCCB 103 isolated from a brackish lagoon, mannitol was the carbon source that allowed both high biomass production and enhanced titers of the antibiotic pyocyanin, a phenazine, which inhibits pathogenic vibrios [41]. 

While the studies described above have all attempted to optimize the production of known metabolites from marine microbes, similar approaches have also been used to produce novel metabolites. In a study by Lin et al., an OSMAC strategy was employed to investigate SM production in the marine-derived fungus *Spicaria elegans* [42]. Earlier work on the strain, isolated from marine sediments in China, revealed the production of cytotoxic cytochalasins when grown on glucose containing media. Lin and co-workers then fermented the strain on 2% starch as the sole carbon source and observed a dramatic shift in SM production, including a novel spicochalasin and 5 novel aspochalasins, in addition to 2 known aspochalasins. The novel spicochalasin was moderately cytotoxic to HL-60 leukemic cell lines and evidence was provided to suggest that it was produced from the same gene cluster which had earlier been shown to produce the cytochalasins following growth on glucose. Overall, this last example underlines the in depth interplay which exits between the regulation of primary and secondary metabolism, which results in significant alteration of the SM profiles. 

### 2.2. Nitrogen Source

The source of nitrogen for both cell growth and SM production is in many ways a more complex consideration than the source of other macro- or micro-nutrients. This is particularly the case if the nitrogen source is amino acid based; as amino acids may play structural roles in NRPS and PKS-NRPS hybrid products or where aminic or amidic moieties constitute part of the structure of the SM. Moreover, as with carbon sources, a balance between cell proliferation and secondary metabolism which often occurs during the stationary growth phase is also an important aspect to consider. This is apparent from a study investigating growth and SM production in the marine-derived fungus *Anthrinium* c.f. *saccharicola* [43]. An inverse relationship was observed between biomass production and the antimicrobial activity of fungal supernatants, when using peptone or yeast extract as the sole nitrogen source in fermentations. High concentrations of the quickly metabolized nitrogen sources favored cell proliferation when the carbon source was limiting and this adversely affected antibiotic production. Conversely, low concentrations of the nitrogen source favored SM production over cell growth [43]. Similarly, nitrogen concentration rather than the nitrogen source itself has been reported to influence the production of anti-cancer and anti-bacterial metabolites in the diatom *Skeletonema marinoi* [44]. Under nitrogen starvation conditions the metabolites produced inhibited the proliferation of human melanoma cells and inhibited the growth of *S. aureus*. These activities were not observed when the diatoms were cultured in nutrient rich media [44]. In agreement with this data, and underlining the effect of the nitrogen concentration on SM production, Wang et al. showed that either very high or insufficient nitrogen concentrations limited the ability of the microalga *Eustigmatos* cf. *polyphem* to produce the antioxidant pigment violaxanthin [45].

A number of the OSMAC studies already described in the previous “carbon source” section also varied the nitrogen sources in their fermentations [38,40,41,42]. In one such study Sujatha and colleagues assessed the effect of 6 inorganic single nitrogen sources and 13 single amino acids, as single nitrogen sources, on the production of the antibiotic SBR-22 in the marine *Streptomyces psammoticus* BT-408 strain [38]. The highest antibiotic yields were observed in fermentations using inorganic nitrates as the sole nitrogen source. In another study anti-MRSA antibiotic production in a marine *Pseudoalteromonas piscicida* strain was examined using media with single nitrogen sources and commercially available media; with antibiotic production being highest when tryptone was the sole nitrogen source [40]. In contrast, in a study examining biomass and pyocyanin production in *Pseudomonas* sp. MCCB-103 where 6 inorganic nitrogen sources, 24 single amino acids, 8 vitamins, urea and casamino acids were assessed as single N sources, urea was found to be the best source for both pyocyanin and biomass production [41]. The effect was quite complex however and was related to the optimum carbon source (mannitol) and to the addition of mineral salts and NaCl [41]. Different nitrogen sources have also been shown to markedly effect cytochalasins produced by the marine-derived fungus *Spicaria elegans*, with spicochalasin A together with five new aspochalasins (M–Q) and two known aspochalasins being produced [42]. Further evidence of the selective regulation of cytochalasins biosynthesis by amino acids was reported when *S. elegans* was shown to produce both novel and known cytochalasins following addition of l- or d-tryptophan to the culture medium [46]. Finally, a novel secondary metabolite, namely elegenketal A, was produced by the marine-derived fungus *S. elegans* when NH_4_Cl was the sole nitrogen source in the fermentation [47]. The aforementioned studies clearly demonstrate that nitrogen sources, be they organic or inorganic, complex or simple have important effects on both biomass and SM production in marine microbes and that sometimes it is the specific carbon:nitrogen ratio or even the synergistic effects of specific carbon and nitrogen sources that are important.

### 2.3. Sulfur and Phosphorus Sources

While the effect of phosphorus concentrations on SM production has been extensively investigated in microbes, the role of sulfur has been less frequently considered. The association between sulfur sources and SMs has been mainly described for sulfur-containing antibiotics produced by terrestrial microorganisms. Studies conducted on β-lactam producing fungi have revealed that the typology of sulfur-containing amino acids affect the induction and titration of the final antibiotic being produced [48]. Similarly, in the actinomycetes *Saccharothrix algeriensis* cysteine and cystine favored the production of sulfur-containing dithiolopyrrolones [49]. It is likely that sulfur-containing amino acids affect the production of these types of antibiotics because they directly provide the sulfur atoms for the final SM [50]. Similar observations have been made in marine bacteria producing the potent antibiotic TDA. The production of TDA and other volatile sulfur-compounds by strains of the *Roseobacter* clade have been reported to be stimulated by the addition of dimethylsulfoniopropionate (DMSP), which is a sulfur-containing compatible solute produced in large quantities by marine algae [51,52].

TDA is also produced by marine bacteria belonging to the *Pseudovibrio* genus [18], and its production was triggered when the strain *Pseudovibrio* sp. FO-BEG1 was cultivation in a defined phosphorous limited medium [53,54]. The effects of phosphorus limitation on the overall cellular machinery regulation and on antibiotic production have been extensively studied in terrestrial actinomycetes [31,55]. Some molecular insights are available and indicate the crucial role of the two-component regulatory system PhoR/PhoB [56]. This system senses the environmental concentration of phosphate, the favored phosphorous source for microorganisms, and directly regulates the expression of some genes responsible for SM production [55]. Similarly, the Pho system interacts with other global regulatory circuits, indirectly affecting the expression of numerous BGCs [31]. A limited number of studies considering the effect of phosphorus concentrations on SM production have been conducted on marine microorganisms. In marine microalgae, it has been shown that phosphate availability influences SM production, and triggers production of antibacterial compounds in some strains [44,57]. Similarly, phosphorous restriction triggers the production of the shellfish-paralyzing saxitoxins in the marine dinoflagellate *Alexandrium minutum* [58]. The type of phosphorous source also influences antibiotic titers in marine bacteria, as a shipworm symbiotic bacterium has been reported to produce the highest amount of the boronated tartrolon antibiotic only when phosphorous was provided in the form of phosphate, and its concentration was maintained at low levels [59]. Despite this, it appears that the manipulation of phosphorous sources and concentrations in the culture media used for marine microorganisms has to date not been extensively or systematically considered. However, considering the drastic impact these conditions have on the SM production in *Streptomyces* and the impact they have on antibiotic production and the overall exometabolome in some marine microbes [53,54,57,60], we believe that this strategy would be a promising route to follow to trigger “silent” BGCs expression in marine microorganisms.

### 2.4. Trace-Elements

The geochemistry of marine environments is quite unique when compared to terrestrial ecosystems, as for example elements such as halogens are abundant in the oceans. Such diversity is mirrored in the peculiarity of many SMs isolated from marine organisms. In fact, while rare in terrestrial organisms, halogenated SMs are common in marine ecosystems, representing most of the 5000 known natural organohalogens [7,61]. Consistently, in recent years, modification of trace-elements concentrations in culture media, such as with the concentration of halogens; has resulted in the discovery of new SMs. Unexpected results were obtained following the addition of 1 mM NaBr to a culture of an *Aspergillus flavipes* strain isolated from wetland mud in Panjin Red Beach, China. This addition led to the production of six known and two unknown cytochalasans, namely flavichalasine N and O [62]. Flavichalasine O was the first example ever described of cytochalasans containing a nitrogen-oxygen heterocycle at the macrocyclic ring part. All these compounds showed strong anticancer properties in in-vitro assays. Potent anticancer molecules were also previously isolated from cultures of the marine fungus *Isaria felina* when the cultures were amended with KBr [33]. Interestingly, the *Isaria felina* strain could produce different halogenated SM depending on the salt composition of the medium. Similarly, the marine fungus *Aspergillus* sp. SCSIO F063 produced seven new chlorinated anthraquinones when grown in sea salt-containing potato dextrose broth. Moreover, when NaBr was added to the medium, two new brominated anthraquinones and one new non-halogenated anthraquinone were produced [63]. Changing the source of halides can lead to the formation of both new molecules and halogenated derivatives of known SMs. For example, the marine fungus *Trichoderma* sp. TPU199, isolated from a red alga, used halide ions from sodium halides in the medium and produced halogenated epidithiodiketopiperazine derivatives [64]. 

Other trace metals have also been shown to both stimulate SM production and activate “silent” BGCs in terrestrial microorganisms [65,66,67]. Similarly, variations in the Mg^2+^ concentration in a defined medium used to cultivate the marine fungus *Ascotricha* sp. ZJ-M-5 resulted in the production of three new caryophyllene sesquiterpenes derivatives, two of which displayed anticancer properties [68]. Such approaches have also been successful in microbes isolated from more extreme environments such as hydrothermal vents. For example, a new anticancer cyclopeptide, clavatustide C, was produced in response to abiotic stress triggered by increased Zn concentrations in a culture of the fungus *Aspergillus* clavatus C2WU, isolated from the hydrothermal vents crab *Xenograpsus testudinatus* [69]. Similarly, stress induced by the addition of metal, especially Ni, triggered the production of new antibiotics in the hydrothermal vent-derived strain *Streptomyces* sp. WU20 [70]. Finally, of particular interest is the effect of rare earth metals, which include the 15 elements from lanthanum (Lt) to lutetium (Lu) plus scandium (Sc) and yttrium (Y). Not only has Sc been shown to enhance actinorhodin production in streptomycetes and bacilysin in *Bacillus*, but, similarly to La, it also triggers the expression of various genes present in otherwise “silent” BGCs [66]. Unfortunately, to the best of our knowledge, no such studies have been performed using marine microorganisms, leaving an as yet underexploited opportunity. 

### 2.5. Comparison between Solid and Liquid Media

The production of SMs has been shown to not only vary with media composition but also differential production has been reported on solid media when compared to liquid media. English and co-workers used a systematic OSMAC approach to test antibiotic production in *Streptomyces* sp. USC-633 isolated from an intertidal environment, examining eleven single carbon sources, with and without added artificial seawater; as well as trace element additions to each formulation and addition of single amino acids. Each media was prepared as a liquid and as a solid medium with the addition of agar [71]. Antibiotic production was assessed by using agar plugs from the solid media and aliquots of liquid culture broths as both ‘spot-in-well’ and paper disk-diffusion assays against lawns of *S. aureus*, *Escherichia coli* ATCC 25922 and ATCC13706, *Enterococcus faecalis* and *Klebsiella pneumoniae*. Antagonistic activities were noted against *E. faecalis* and *E. coli* ATCC 13706 from the agar plugs but activity from the liquid broths (both spot-in-well and paper disk-diffusion assays) was only observed against *E. coli* ATCC 13706. Subsequently, organic extracts from agars where activities were observed were prepared and fractionated for chemical analyses by NMR. When comparing these extracts to the corresponding liquid broth extracts (where no antibiotic activity was detected) different NMR spectra were noted with compounds in the “active” agar extracts being absent from the “inactive” liquid broths. Comparable differences were also observed by Liu and colleagues, who noted different SM profiles from the sponge-derived fungus, *Penicillium adametzioides* AS-53 when grown in liquid (potato-dextrose-broth) and solid (rice solid culture medium) media [72]. The organic extracts from the former yielded a novel spiroquinazoline (N-formyllaptin A), novel bisthiodiketopiperazines (adametizines A and B), and several known meroterpenes. Extracts from the solid media fermentation yielded novel acorane sesquiterpenes (adametacorenols A and B) as well as dimethyl-N-(phenylacetyl)-glutamate which were not seen in the liquid fermentation extracts. Adametizine A displayed cytotoxic activity in a brine shrimp lethality assay and subsequently displayed selective cytotoxic effects against one of fourteen cancer cell lines tested. Additionally, adametizine A and B displayed broad range antimicrobial effects. Similarly, during OSMAC based experiments conducted by Guo and co-workers using the deep-sea-derived fungus *Penicillium* sp. F23-2, it was observed that switching from complex liquid media to a rice-based solid medium triggered a drastic change in the SMs produced by the strain. Cultivation using the solid medium led to the isolation of five new ambuic acid analogue (penicyclones A−E), which showed antibacterial activity against *S. aureus* [73]. It is important to clarify that in the latter examples it is unclear whether the changes in metabolite production were caused by the growth on solid media, or by the different chemical compositions of the complex media used. In fact, different complex media can often have drastic effects on microbial SM production. For example, the SM profile of the deep-sea sediment-derived strain *Streptomyces* sp. YB104 showed significant variations when the strain was cultivated in seven different complex media [74]. Nevertheless, it is clear from the work of English et al. [71] that further investigations into the effect of solid versus liquid media on SM production are warranted.

## 3. Change in Physical Parameters

During the taxonomic characterization of newly isolated microorganisms, it is a common practice to test optimal growth conditions, including primarily temperature, salinity, and pH. These and other factors that do not directly concern the nutrient composition of the media can have drastic effects on the growth dynamics of bacteria, and they can also have marked effects on SM production. In their study postulating the OSMAC principle, Zeeck and colleagues were able to discover >10 additional metabolites produced by *A. ochraceus* by changing cultivation parameters such as salinity, temperature, and even vessel types [26]. Similar results have also been observed in marine microorganisms, as for example in a marine *Alteromonas* strain which grows best at 28 °C and produces the highest levels of an antiviral SM when grown at 25 °C [75]. Another well-known example is the effect of shaking conditions on the production of TDA by marine strains of the *Roseobacter* clade, with the potent antibiotic mainly being produced when the strains are growing under static conditions [52]. 

### 3.1. Temperature

The temperature used for growth effects both cell proliferation rates and secondary metabolism. For example, Miao and colleagues reported that *A. saccharicola* grew faster at 30 °C but that SM production by that fungus was highest at 25 °C [43]. Similarly, Darabpour et al. showed the effect of temperature on the production of an anti-MRSA antibiotic by *Pseudoalteromonas piscicida* PG-02. The authors tested seven different temperatures and showed that 28 °C was the optimal temperature for maximizing antibiotic titers [40]. An interesting study conducted using diatoms showed that molecules with different bioactivities were preferentially produced under different temperature ranges. In this study five species of diatom were cultivated under two different illumination and temperature regimes and the production of molecules with anti-oxidants, anti-diabetes, anti-cancer, immunomodulation and anti-infection activities was monitored [76]. Each species was grown at different high and low temperatures, ranging from 3.3 °C–5.6 °C (low) to 6.6 °C–9.0 °C (high) which were determined in pilot scale experiments. The numbers of “hits” against the five assay categories under the four cultivation regimes (low light-low temperature; low light-high temperature; high light-low temperature; high light-high temperature) varied by species and by assay category. For example, bioactivities from *Attheya longicornis* were only noted in extracts from high temperature cultivations. The only bioactivity from *Chaetoceros furcellatus* was observed from extracts produced in low temperature conditions. Extracts from *Chaetoceros socialis* showed anti-cancer and anti-diabetes activities only when grown at high temperatures but only displayed anti-oxidant activities from low temperature cultivations. *Porosira glacialis* displayed anti-cancer and anti-diabetes activities under both high and low temperatures but showed anti-oxidant activities only when grown at low temperatures. Finally, *Skeletonema marinoi* displayed anti-oxidant activities under both low and high temperature conditions but anti-cancer activities were only observed following cultivation at high temperature. Another interesting example is the fungal strain *Penicillium raistrickii* JH-18, isolated from a coastal marine saline soil, which was able to produce five new polyketides, namely raistrickiones A–E when the fermentation temperature was shifted from 28 °C to 15 °C [77]. However, in other cases specific metabolites have been shown to be produced over a wide range of temperatures. For example, Sanchez and co-workers observed that the peptide antibiotic andrimid was produced by the psychrotolerant strain *Serratia proteamaculans* over a range of temperatures from 4 to 20 °C [78]. Low titers of the antibiotic were also detected in cultures growing at 25 °C, with no production being observed at higher temperatures. 

### 3.2. Vessel Types, Aeration, and Shaking Conditions

Recent studies have shown that the design of fermentation vessels, particularly where niche mimicry allows for biofilm formation; can have a marked effect of SM production in marine microbes. While it is accepted that the true ecological role of many microbial SMs has not been experimentally validated, antibiotic production has long been believed to be involved in competitive inhibition of the growth of proximal microbes or possibly in intercellular signaling. The Mukherjee group reported several findings connecting vessel types, lifestyles, and SM production in various microorganisms [79,80,81]. For example, Mitra and colleagues designed a conico-cylindrical flask (CCF) with an ultra-low-speed rotating disk bioreactor [79]. The disk rotated one revolution per day, was submerged for 12 h and above the liquid media for 12 h as a niche-mimic strategy to replicate the intertidal environment from where a *Shewanella* sp. was isolated. This elegant design was seen to enhance biofilm formation by the strain and also resulted in enhanced production of melanin. In an earlier study the same group monitored fermentations of *Shewanella colwelliana* (for melanin production) and *Pseudoalteromonas rubra* (for antibiotic production) in 3 vessel types; (i) CCF with a hydrophobic (polymethylmethacrylate [PMMA]) attachment surface, (ii) CCF with a hydrophilic (glass) attachment surface and (iii) a standard unbaffled Erlenmeyer flask (EF) [80]. By monitoring growth rates, biofilm formation, and SM production they reported a positive correlation between reactor surface area, hydrophobicity, cell growth, biofilm formation and melanin production in *S. colwelliana*. In contrast, although cell growth and biofilm formation by *P. rubra* were highest in the CCF-PMMA configuration, highest antibiotic activities were observed from the EF fermentation. These findings highlight that bioprocess intensification is a strain-specific endeavor and that it is necessary to investigate variations in the scale of bioreactor design, before scale-up bioprocesses are undertaken. The Mukherjee group have continued to exploit their patented CCF-PMMA bioreactor, most recently to investigate metabolite production profiles of three intertidal cyanobacterial isolates, namely *Oscillatoria* sp. AP17, *Leptolyngbya* sp. AP3b, and *Chroococcus* sp. AP3U [81]. Upon comparing the metabolite profiles of these strains, grown in biofilms in the CCF-PMMA, to those of the same strains grown as plankton in a standard EF; they found that biofilms formed in the CCF but not in the EF and that cell-bound polysaccharide and released polysaccharide production was higher in CCF than in EF. Furthermore, they reported greater antimicrobial activities from cyanobacterial extracts grown in CCF when compared to extracts from the EF grown cultures. 

The typologies of the cultivation vessels can also affect aeration within bioreactors, which has long been used to stimulate microbial growth and to correspondingly increase SM production. Generally, the aeration method involves shaking vessels or bubbling air through a submerged sparger to increase dissolved oxygen levels and to replace depleted oxygen. However, in a study by Burja and co-workers, increased lipopeptide production was observed in the cyanobacterium *Lyngbya majuscula* when air was circulated within the reactor vessel above the water level, and no agitation of the vessel to increase dissolved oxygen levels was employed [82]. The effect of oxygen concentration on SM production was clearly demonstrated in a recent study conducted on the marine-derived *Streptomyces* sp. CNQ-525 [83]. In this study, hypoxia triggered a shift from production of the antibiotic napyradiomycin to production of 8-aminoflaviolin, an intermediate in the napyradiomycin biosynthetic pathway. 8-aminoflaviolin was shown to be a redox active compound, which the authors hypothesized might function as an extracellular electron shuttle [83]. In batch experiments aeration is often controlled via regulating the shaking speed, and this has also been shown to affect SM production, as for example in the above-mentioned example of TDA production within the Roseobacter clade [52]. In another study a strain of the marine-derived fungus *Tolypocladium geodes* (strain MF458) was investigated for the production of bioactive SMs following fermentation in six different media compositions [84]. A suite of cytotoxic compounds was produced including known *Tolypocladium* spp. metabolites (cyclosporine A and efrapeptins), a metabolite previously isolated from *Acremonium* spp. (pyridoxatin), a previously known fungal cytotoxic metabolites (malettinins B and E, tolypocladenols A1 and A2) and the novel compound tolypocladenol C. Optimal production of most of these compounds occurred under standing (non-shaking) fermentation conditions that allowed the formation of a ‘mycelium cake’. Contrastingly, switching from static to shaking cultivation conditions resulted in the above mentioned deep-sea-derived fungus *Penicillium* sp. F23-2 producing five new nitrogen-containing sorbicillinoids named sorbicillamines A–E [85]. These studies demonstrate that the design of a bioreactor vessel and the aeration together with the agitation regimes can all have an impact on the production of secondary metabolites; but crucially, not in an identical fashion for all microbes so species- and strain-specific optimization is necessary in each individual fermentation to maximize production of the metabolite(s) of interest.

### 3.3. Osmotic Stress, Salinity, pH

Marine microbes inhabit a variety of different environmental niches which are governed by different physico-chemical conditions. For example, sea water itself is a mixture of 96.5% water and 3.5% other materials, including salts, dissolved gases, organic substances, and undissolved particles, with 87% of dissolved salts being NaCl. Ocean salinity is approximately 3–3.5% which can increase in hypersaline micro-environments such as brine pools or decrease in mangroves. Thus, it is reasonable to presume that expression of marine-associated microbial metabolites has evolved to best adapt to a variety of these conditions. One of the first reported examples of how salinity affects SM production described how among four actinomycetes isolates only one isolate maintained an ability to synthesize bioactive SM in the absence of seawater [5]. Another example of the effect of salinity on SM production was reported by Saha and colleagues [39]. They monitored the production of an antimicrobial lipid from a marine-derived actinobacterium, assessing the growth at different salinities using both natural and artificial seawater containing only NaCl as salt. While the isolate could grow in up to 20% NaCl, antibiotic production ceased at a concentration between 5% and 10% NaCl. Effects as the one mentioned above have been directing researchers to mimic marine conditions in bioprocesses to enhance SM production. In this respect, the collection of relevant metadata from isolation sources should not be underestimated if appropriate conditions are to be replicated to encourage SM production. 

Overy and colleagues investigated the effects of osmolarity and salinity on the production of SMs in the terrestrial halophilic fungus, *Aspergillus aculeatus* [86]. They found that SMs such as aspergillusol, secalonic acid D, aculene C and another aculene analog were up-regulated in the fungus under saline conditions; with production of aspergillusol in particular being associated with a halotolerant response to saline conditions involving seawater/sea salts. They concluded that the addition of seawater or salts to the fermentation medium of halotolerant fungi is likely to result in an increase in SM production. Similarly, high salt stress has been reported as a determining factor in the production of novel antimicrobial compounds produced by the marine sediment-derived fungus *Aspergillus terreus* PT06-2 [87]. When this strain was grown in a range of different salt concentrations twelve known compounds and three new metabolites (terremides A and B and terrelactone A) were produced. Growth at the highest salt concentration resulted in a higher level of overall SM production and specifically induced production of two of the new metabolites. Similarly, growth under high salt stress conditions in the marine-derived fungus *Spicaria elegans* resulted in the production of six compounds, including one novel compound, (2E,2′Z)-3,3′-(6,6′-dihydroxybiphenyl-3,3′-diyl) diacrylic acid which displayed mild antimicrobial activity against *Pseudomonas aeruginosa* and *E. coli* [46]. In contrast low salinity, has been reported to represent a stressful condition in the marine bacterium *Salinispora arenicola*, and changes in salinity concentration had different effect on different types of SMs produced by this bacterium [88,89]. 

Finally, it is worth mentioning the effect of pH on SM production. In general, marine environments are slightly alkaline in nature, thus pH monitoring and adjustment is advisable when marine microbes are used for NPs discovery. Sakar and co-workers have reported the effect of pH on SM production, with antibiotic production in a marine *Streptomyces* sp. being influenced by biofilm formation and that biofilm formation correlated with the pH of the culture medium [90]. When testing culture broths for antimicrobial activities it was noted that no activities were present from an acidic medium (pH 4.0) but activity was seen at up to pH 10.0, being highest at pH 9.0. Similarly, Darabpour et al. reported that slightly alkaline pH was optimal for the production of anti-MRSA antibiotic by *Pseudoalteromonas piscicida* PG-02 [40].

## 4. Co-Cultivation and Other Environmental Cues

Surely one of the most promising and exciting techniques being employed to stimulate the production of SMs and the activation of “silent” BGCs is the co-cultivation of different microorganisms. Even though co-cultivation strategies were not directly considered when the OSMAC approach was originally conceptualized [26], we believe that they can be considered as a natural extension of this approach. In nature microbes not only face changes in both physical and chemical abiotic parameters, but they are also involved in a dynamic network of intra- and inter-species interactions. These interactions can be driven by diffusible molecules or require direct contact between cells, and can influence the cellular machinery initiating processes aimed at increased competitiveness and fitness that often translate into the production of bioactive SMs. Seminal observations, made over 30 years ago, reported an increase in antagonistic activities in different terrestrial fungal strains when they were co-cultivated [91,92,93]. Such effects relied on the production of various SMs sensed by the neighbouring strain and eliciting in it a strong increase in the production of anti-fungal compounds [92]. These findings were then extended by the Fenical group that isolated the new antibiotic pestalone from a culture of a marine fungus challenged with a bacterial strain [94]. Based on the same interaction principle other strategies relying on the use of chemical cues, such as sub-inhibitory antibiotic concentrations, were tested in the last number of years. Altogether these cultivation procedures have been increasingly employed, and today represent highly promise rout for SM discovery. Usually, the co-cultivation of either different bacterial and/or fungal strains is employed, but recently, it has been shown that the addition of bacteriophages can also have an effect on SM production. This phenomenon was described in a deep-sea hydrothermal vent-derived *Geobacillus* sp. E263, which produced a novel anti-tumor quinoid only when challenged with the bacteriophage GVE2 [95].

### 4.1. Prokaryote-Prokaryote Co-Cultivation

Co-cultivation experiments have been performed either with distantly related bacterial species or with co-occurring or co-specific bacterial strains. A good example of the latter approach was recently reported where different strains of the freshwater cyanobacterium *Microcystis aeruginosa* changed their SM profiles producing cyanopeptolins, aerucyclamides and aeruginosins, in response to intra-specific co-cultivation [96]. In addition, the unusual strategy of co-cultivating marine bacteria with human pathogenic bacteria also led to interesting results, with *S. aureus*, *P. aeruginosa*, *E. coli*, *Bacillus* spp. stimulating marine bacteria to produce antibiotics, biosurfactant, and quorum-sensing inhibitors which were active against the challenging strains [97,98,99]. It is interesting to note that such induction was present not only when strains were co-cultured, but also when the two cultures were physically separated by a semi-permeable membrane, suggesting that diffusible molecules were involved in eliciting production of the SMs [97]. In another study Fdhila and coworkers isolated new dd-diketopiperazines, which strongly inhibited the marine pathogen *Vibrio anguillarum*, from two epibiontic bacteria when co-cultured with *V. angiullarum* [100]. Co-cultivation of marine bacteria isolated from similar habitats has also resulted in altered SM profiles, with new or higher titers of NPs being produced when compared to monocultures [101,102]. Specifically, when the sponge-derived actinomycetes *Actinokineospora* sp. EG49 and *Nocardiopsis* sp. RV163 were co-cultured, they produced three NPs that were not detected in single cultures of either bacterium [101]. Similarly, a *Bacillus* sp. (UA-094) isolated from the seaweed *Ulva californica* produced indole and the diketopiperazine cyclo (Phe-Pro) at higher levels when challenged with another *Bacillus* strain [102].

Perhaps, one of the most relevant and widely investigated phenomena is the effect of mycolic acid-containing bacteria on SM production in actinomycetes. In a screen aimed at identifying soil bacteria that were capable of triggering SM production in streptomycetes, Onaka and colleagues found that several mycolic acid-containing bacteria such as, *Tsukamurella, Nocardia, Rhodococcus, Mycobacterium*, and *Corynebacterium* triggered production of the two red pigments actinorhodin and undecylprodigiosin [103]. Surprisingly, the authors showed that the simple addition of mycolic acid into the medium did not trigger pigmentation production, but a physical contact between the cells was needed. This mechanism is different from the one involving signaling via diffusible molecules such as the A-factor and goadsporin, which are produced by actinomycetes species and which can induce SM production within this bacterial group [103]. The induction of SM production by the addition of mycolic acid-containing bacteria is common among streptomycetes, as evidenced by the fact that 99 out of 112 tested strains displayed altered SM profiles when exposed to mycolic acid-containing bacteria; resulting in the isolation of several novel bioactive SMs in terrestrial streptomycetes [103,104]. Unfortunately, to date, this approach has been poorly explored within marine actinomycetes, even though metabolomic data underlined the dramatic impact such combinations had on the SM profiles of marine Micromonosporaceae [27]. Finally, an interesting strategy was recently used with samples collected from Colombian solar salterns, which were used to create enrichment cultures from which metabolites were extracted and tested for antibacterial and anticancer activities [105]. This approach did not combine previously isolated strains but used a more direct strategy streamlining the discovery of various bioactivities. Such technique represents an easier and faster method to select bioactive consortia, which can then be characterized using both cultivation-dependent and independence methods.

### 4.2. Prokaryote-Eukaryote Co-Cultivation

The most successful co-cultivation approaches which have been undertaken to date, arguably involve various fungal strains. We have already described the isolation of the potent antibiotic pestalone, which was produced by a fungus of the *Pestalotia* genus, isolated from the brown alga *Rosenvingea* sp., only when co-cultured with a marine bacterium [94]. The latter was then characterized as belonging to the *Thalassospira* genus, and it was subsequently shown that the same bacterial strain also triggered the production of four new anticancer diterpenoids, namely libertellenones A–D, when added to a culture of the marine-derived fungus *Libertella* sp. [106]. Similarly, two new cyclic depsipeptides, emericellamides A and B, where isolated from a culture of the marine fungus *Emericella* sp. when co-cultured with the marine actinomycete *S. arenicola* [107]. Other examples of the effects that co-cultivation with bacteria has on fungal SM profiles, include the isolation of 10 new and 13 known prenylated 2,5-diketopiperazines, following co-cultivation of the marine fungus *Penicillium* sp. DT-F29 and the bacterium *Bacillus* sp. B31 [108]. Similarly, co-cultivation of *Streptomyces fradiae* 007 and *Penicillium* sp. WC-29-5 led to a shift in the SM profiles of the strain and resulted in the isolation of two new polyketides with anticancer properties [109]. 

Such effects can also be bi-directional, as bacteria can respond to the presence of fungal strains with the production of various SMs. In fact, metabolomic studies have shown dual induction of bacterial and fungal metabolites by the co-cultivation of the marine fungus *Aspergillus fumigatus* MR2012 and two hyper-arid desert *Streptomyces leeuwenhoekii* strains, leading to the isolation of the new compounds luteoride D and pseurotin G [110]. In another study Moree et al. showed that the side-by-side growth of a *Bacillus amyloliquefaciens* strain isolated from an octocoral and an *Aspergillus* strain enhanced the bacterial production of antifungal compounds which were identified as novel members of the iturin family [111]. Mechanistically, it was reported that streptomycetes can trigger modification of fungal histones and in turn elicit BGCs activation, and in some cases a direct contact between the microbes is needed [112,113]. In the aforementioned example regarding libertellenones, physical contact between the bacterium and the fungus *Libertella* sp. was also required for the production of the diterpenoids, as no production was observed either in *Libertella* monocultures or following addition of the bacterial culture supernatant. Most surprisingly, molecular interaction mechanisms can in some cases result in a bioactive metabolite which is produced as a result of the fungal modification of a metabolite initially produced by the bacterium, as in the case of *Rhizopus microsporus* and its endosymbiotic bacterium *Burkholderia rhizoxinica* [114]. 

### 4.3. Eukaryote-Eukaryote Co-Cultivation

Co-cultivation of eukaryotic species has also been reported to elicit SM production. In a study by Chen and colleagues two *Penicillium* spp. isolates (strains IO1 and IO2) from the marine sponge *Ircinia oros* were co-cultured [115]. Mono-cultures of these strains yielded known compounds, griseofulvin and dechlorogriseofulvin from IO1 and dehydrocurvularin and curvularin from IO2 as well as a new fusarielin derivative from IO1. Co-cultivation, however, induced production of the known SMs norlichexanthone and monocerin which were not observed in the mono-culture fermentations. Cytotoxic activities were observed from dehydrocurvularin and from monocerin against the L5178Y mouse lymphoma cell lines. The authors hypothesized that because norlichexanthone and monocerin had previously been reported as antibacterial and antifungal compounds their production under co-culture conditions may have been induced as a stress response aimed to suppress the growth of the competitor strain. The production of derivatives of known compounds has also been reported following co-culturing experiments. When the marine sediment fungal isolate *Aspergillus sulphureus* KMM 4640 and another marine sediment fungal isolate *Isaria felina* KMM 4639 were co-cultured, not only were four known diorcinols (B–E) produced, but a new derivative diorcinol J was also discovered [116]. Other fungal based co-culture experiments involving *Chaunopycnis* sp. CMB-MF028 and *Trichoderma hamatum* CMB-MF030, both isolated from an intertidal mollusk *Siphonaria* sp., resulted in the production of a rare class of 2-Alkenyl-Tetrahydropyran, namely chaunopyran A, and methylated analogues of the pyridoxatins [117]. *Chaunopycnis* sp. CMB-MF028 which is known to produce pyridoxatin and tetramic acids was co-cultured with five other fungal strains which had been co-isolated from the same mollusk, but interestingly, only co-culture with *Trichoderma hamatum* CMB-MF030 yielded new chemistry.

While many co-cultivation experiments involve fungal strains isolated from the same or similar marine habitats, using the premise that the strains involved are likely to be in natural competition with each other, co-cultures of fungi from very different environments have also been performed. For example, a deep-sea derived species *Talaromyces aculeatus* was co-cultured with a mangrove isolate *Penicillium variabile* HXQ-H-1 [118], and this resulted in the production of not only one known polyketide, nafuredin A, but also of four novel polyketides, nafuredin B and pentitalarins A–C; none of which were observed in mono-culture experiments. Importantly, nafuredin B was found to display cytotoxic activities against a range of cancer cell lines. A different co-culture approach was used by Mandelare and co-workers, who used two different morphological, developmental stages of the alga-derived fungus, *Aspergillus alliaceus* [119]. While the *A. alliaceus* sclerotial morph produced ochratoxin and the vegetative morph produced an anthraquinone pigment nalgiovensin, the co-culture of these morphs resulted in a marked change in metabolite profiles, with production of a chlorinated congener of nalgiovensin, nalgiolaxin and allianthrones A–C. In cytotoxicity assays allianthrone A showed weak activity against HCT-116 colon cancer and SK-Mel-5 melanoma cell lines. The eukaryote-eukaryote co-cultivation experiments described here have shown imaginative experimental designs and have elicited the production of SMs with interesting bioactive properties. These clever approaches may inspire others to undertake similar studies and also to employ novel experimental parameters to allow the identification of new chemical entities from marine sources.

### 4.4. Addition of Chemical Elicitors 

Many of the microbial interactions that elicit the production of bioactive SMs are underpinned by complex chemical ecology mechanisms relying on diffusible molecules. Some of these molecules are produced by con-specific of close taxonomic relatives. Classical examples of such mechanisms are autoinducer molecules that modulate quorum sensing communication processes. Autoinducers are generally used to recognize population cell density while in turn regulating various cellular processes, amongst which is antibiotic production [74]. Quorum sensing-like regulation relies on the concentration of the autoinducer in the environment. When the population cell density increases, the extracellular concentration of produced autoinducers increases as well, with these molecules then being sensed by the neighbouring cells triggering gene regulation. This type of regulation has been reported in various bacterial groups, and affects the production of different type of bioactive SMs such as polyketides, β-lactams, and phenazines [13,120,121]. In theory the effectiveness of the antibiotic is maximized if the entire bacterial population concomitantly produces and releases the molecule(s), thereby increasing its concentration in the immediate environment. Well known examples of molecules which regulate SM production came from the actinomycetes group, and include the autoinducer A-factor and the γ-butyrolactone, which have been shown to have a broad impact on SM production in these bacteria. Similarly, the PI-factor, or 2,3-diamino-2,3-bis(hydroxymethyl)-1,4-butanediol, and a recently discovered hydroxymethylfuran signalling molecule have both been shown to activate production of antibacterial and antifungal compounds in streptomycetes [103]. In some cases, the regulation may also be negative, as in the rice paddy isolate *Burkholderia thailandensis* where deletion of a LuxR-type quorum sensing transcription regulator, which abolished the quorum sensing regulation, elicited production of the novel polyketide thailandamide lactone [122]. Even though autoinducers are in general produced by the same bacterial population which undergo SM production regulation, there are examples in both marine and terrestrial bacteria in which the quorum sensing response, and hence SM production, is altered by autoinducer-like molecules produced by other bacterial strains. For example, the marine Gram-positive bacterium *Halobacillus salinus* produces phenethylamide which has been shown to alter violacein production in *Chromobacterium violaceum*, in a quorum sensing-like fashion [123]. Similarly, exogenously produced diketopiperazine molecules are known to be capable of activating or antagonizing some quorum sensing-regulated mechanisms and have been shown to induce the marine sponge-derived strain *Pseudoalteromonas* sp. NJ6-3-1 to produce antibiotics at low cell densities [124]. Recent genomic analyses showed that LuxR-type transcription regulators, which are able to sense the acyl homoserine lactone autoinducer and regulate gene expression, are often associated with Proteobacteria BGCs [125]. This suggests that these LuxRs, and thus quorum sensing, might affect the transcription regulation of these BGCs. Moreover, some bacterial groups have been shown to encode only the systems required to sense external presence of autoinducers [126,127]. For example, the marine Alphaproteobacterium genus *Pseudovibrio*, which possesses potential for novel SM discovery [17,18], harbours strains that encode both the sensing and producing systems, and strains that are only able to sense the presence of autoinducers [17,127]. Taken together this data suggests that the addition of externally produced autoinducers may represent a promising exploration route for triggering SM production in marine bacteria, even though to date this approach has been largely overlooked. 

Two other broad groups of chemical cues with marked effects on bioactive SM production in bacteria and fungi are antibiotics and epigenetic modifying compounds. An in-depth analysis of numerous examples covering these phenomena can be found in a recent comprehensive review by Okada and Seyedsayamdost [128]. With respect to antibiotic production, it is known that bacteria often respond to potential antibacterial threats by producing antibacterial SMs. For example, the aforementioned goadsporin is an antibiotic produced by certain *Streptomyces* strains, but at lower concentrations (<1 μM) elicits the production of SMs in various streptomycetes [103]. The ability to stimulate SM production at low dosage and inhibit cell proliferation at high dosage has been observed for several antimicrobial compounds effecting a diverse range of microorganisms. For example, promomycin, triclosan, ARC2 and its derivative Cl-ARC2 elicit the production of novel SMs in a wide variety of streptomycetes [128]. These effects can be dramatic, as shown by the fact that the addition of Cl-ARC2 to cultures of multiple *Streptomyces* strains induced production of 216 cryptic metabolites [129]. Similarly, in a recent high-throughput screen aimed at finding chemicals eliciting bacterial SM production, it was reported that the majority of elicitors were antibiotics, which triggered SM production at sub-lethal concentrations [130]. Amongst these, trimethoprim was found to act as a global secondary metabolite activator, eliciting the expression of multiple “silent” BGCs in *B. thailandensis* [128]. Even though these modifications of cultivation conditions are highly promising, they have to date been almost completely overlooked in marine bacteria. The potential of these approaches within marine microbes is underlined by the pleiotropic regulatory effect of the potent antibiotic TDA in the marine bacterium *Phaeobacter inhibens*, in which the addition of TDA at concentrations 100-fold lower than the MIC impacted expression of ~10% of the total genes in the strain, having regulatory effects similar to the canonical quorum sensing systems [131]. Therefore, altogether these data call for a more systematic effort directed to the use of such approaches on talented marine microorganisms.

Molecules that alter epigenetic regulation in fungi, especially via chromatin remodeling, belong to the second group [112,132]. The reason behind such effects is easily envisioned, as remodeling of the chromatin would loosen protein-DNA complexes, making the DNA more accessible to the transcriptional machinery in the cells. Given that BGCs in fungi are often clustered together, then chromatin remodeling can have a marked effect on SM production [132,133]. This approach has already proven its potential when applied to marine fungi. The marine sediment-derived fungus *Microascus* sp. was able to produce a new cyclodepsipeptide called EGM-556 when cultivated in the presence of the histone deacetylase inhibitor suberoylanilide hydroxamic acid (SAHA) [134]. Similarly, cultivation of the marine fungus *Penicillium variabile* HXQ-H-1 in the presence of the DNA methyltransferase inhibitor 5-azacytidine led to the discovery of a new highly modified fatty acid amide varitatin A which has anticancer properties [135]. When the same strain was then cultivated in the presence of SAHA, seven polyketides were produced, including four new varilactones A–B [136]. Similarly, histone deacetylase inhibitors added to the culture of the marine fungus *Cochliobolus lunatus* (TA26-46) resulted in a significant change in the SM profile with production of two unknown 14-membered resorcylic acid lactones having a bromine substitution, a modification which until then had not previously been observed in these type of molecules [137]. Besides chemicals that interfere with chromatin remodeling, additional compounds influencing fungal cellular machinery have been shown to elicit cryptic SM production. For example, addition of the F-actin inhibitor jasplakinolide to the cultivation medium of the marine fungus *Phomopsis asparagi* led to production of three new SMs, namely chaetoglobosin-510, -540, and -542, which displayed cytotoxic bioactivity against murine colon and leukemia cancer cell lines [138]. 

Finally, we would like to point out an additional strategy that does not use specific chemicals, but uses bioorganic compound mixtures. This can be considered as a direct extension of the co-cultivation approaches, as for example the homogenized cell wall of fungi has been shown to increase SM production in both cyanobacteria and streptomycetes. In some cases, the elicitors were characterized as chitin (or N-acetylgucosamine) derivatives of various length [128]. Similarly, as mentioned earlier, chitin, a polymer abundant in marine invertebrates, has been reported to increase the production of the hybrid non-ribosomal polyketide peptide andrimid in the putative coral pathogen *V. coralliilyticus*, and it has been suggested that this increased production might represent an adaptation of *Vibrio* spp. aimed at increasing their competitiveness when sensing compounds indicating the presence of potential hosts to colonize [35]. In the last number of years, host-associated microorganisms have been an important source of novel bioactive SMs, as unquestionably exemplified by sponge-associated microorganisms [18,139,140,141]. Mechanistically, the association between host and microbes relies on bi-directional interactions based on both direct cell-to-cell contact and diffusible molecules. Therefore, it is likely that homogenized tissue or metabolite extracts from the animal host will contain chemicals that are capable of influencing microbial cell machinery and hence SM production. This assumption is based on the fact that the marine animal host and associated microbes have co-evolved, developing interaction strategies aimed at maximizing their fitness. For example, microbes can protect hosts from parasites or pathogens, while the host provides the associated microbes with metabolic intermediates, decreasing the metabolic costs faced by the microorganisms [142]. These approaches have rarely been considered, and we believe that future efforts directed to the discovery of novel marine NPs should be guided by an increased consideration of the chemical ecology underpinning such associations, which may ultimately lead to the discovery of molecules with novel bioactive functions.

## 5. High Throughput Methods to Streamline Cultivation Based Biodiscovery

One of the main drawbacks of the cultivation-based approaches described above is the fact that due to their empirical nature they are quite labour intensive. In other words, extensive laboratory experiments are needed to verify which modifications in the cultivation practices elicit the synthesis of cryptic metabolites. However, several strategies have been developed to help streamline the testing processes. The focus of this review dictates that we will not cover the use of omics-based approaches to identify BGCs, and we refer the reader to a number of recent relevant reviews in this area [12,13,15,143]. Following identification of a strain which based on preliminary experiments or genomic data is considered “talented”, several experiments need to be performed to account for biological variability and various cultivation conditions. The need to increase the experimental throughput resulted in the development of micro-fermentations (Figure 1), in which the cultivation volume is considerably reduced allowing highly parallelized cultivation, decreased set-up times, and even the automated control of cultivation parameters [144,145]. Various types of bioreactor setups are currently available, ranging from the simple microtiter plate-based systems using <1 mL volume to bioreactors of a few mL that permit continuous cultivation [144]. These approaches have been used with both bacteria and fungi, allowing for example, the growth of 44 marine Microccocaceae using a wide array of nutrients, and leading to the generation of 528 crude extracts. From these extracts the new anti-MRSA compound kocurin, a new member of the thiazolyl peptide family, was isolated [146]. Similarly, micro-fermentation-based studies have also been used to evaluate the ability of mycolic acid-containing bacteria to induce SM biosynthesis in marine invertebrate-associated Micromonosporaceae [27]. Co-culture was conducted in 500 μL volumes and, following incubation, micro-extractions were performed and crude extracts were used for LC-MS analyses and for high-throughput agar-based bioassays [27]. These methods can greatly help in streamlining the initial stages of the biodiscovery process when multiple cultivation conditions need to be analyzed. Similarly, a combination of microfermentation and genetic manipulation have been used to develop a high throughput method to verify the suitability of an array of chemicals as elicitors of SM production. In this method, a lacZ translational fusion to an essential gene within a selected BGC is created and used as a reporter system [130]. The engineered strain is then grown in microtiter plates in the presence of an array of potential chemical elicitors. Increased lacZ activity indicates that the elicitor induces expression of the gene within the BGCs. Such approaches have led to the discovery that sub-lethal concentrations of nine antibiotics trigger SM production in *B. thailandensis*. Although only applicable to microbial strains that are amenable to genetic manipulation, this is an intelligent approach that allows problems associated with low titers of SMs to be circumvented. We believe that based on micro-fermentations, alternative approaches, so far unexplored, could be developed building on the aforementioned work of Conde-Martinez et al. [105]. After collecting environmental samples, enrichment cultures can be obtained using an array of growth conditions with micro-fermentation devices. The cultivation of the enrichments displaying the most interesting bioactivities can then be scaled-up and the enrichments can be further characterized. These latter approaches will also help to circumvent the problem of obtaining single microbial strains, which in some cases can be difficult to isolate in monoculture.

Within the OSMAC approaches, optimization of the cultivation conditions can be achieved by simply modifying one-factor-at-a-time, or can be based on more sophisticated mathematical and statistical approaches that can help to more efficiently and effectively identify the correct parameters to manipulate [147]. The classical one-factor-at-a-time approach has been widely used in industrial microbiology, and involves modification of a single cultivation parameter at a time, for example, the source of carbon over an array of concentrations. This probably represents the first type of screening which needs to be performed when little or no information about the microorganism and the media in which they are likely to grow and produce SMs is available. In contrast, mathematical and statistical approaches can allow a decrease in the number of variables tested, reducing the processing time and the labour costs. Generally speaking, such approaches use the data obtained from preliminary experiments where only a few parameters, which among all potential variable conditions are more likely to induce a certain process or production of a product, are evaluated (Figure 1) [147]. Experimental design, which is the general name used for such practices, or statistical techniques derived from it, i.e., response surface methodology (RSM), have been successfully used to increase production of various bioactive SMs from both marine bacterial and fungi. Examples of where RSM has been successfully employed include optimization of antibacterial, antifungal, and antioxidant activities in the deep sea-derived marine actinomycete *Streptomyces sparsus* VSM-30; allowing the subsequent identification of a variety of diverse SMs in ethyl acetate extracts [148]. In addition, RSM has been used to optimize antimicrobial metabolite production by up to 3.86-fold, in *Micromonospora* sp. Y15 a sea mud isolate from the South China Sea [149]. With respect to marine fungi, RSM has also been employed to optimize culture conditions for the production of penicilazaphilone C, a new antineoplastic and azaphilone from the marine-derived fungus *Penicillium sclerotiorum* M-22 [150]. While useful in the optimization of media composition and increasing production of SMs, the aforementioned experimental designs can be challenging to apply at the beginning of the biodiscovery work-flow, when the metabolites of interest have not yet been identified. However, we envision that such approaches might be particularly useful when novel metabolites with a desired action are being targeted. For example, instead of evaluating which conditions are more likely to enhance the production of a specific SM, these approaches could instead be used to find the optimal growth conditions that maximize a specific bioactivity; such as inhibition of MRSA. Thus, by combining these experimental designs with a rational selection of medically-relevant bioactivities, the detection of pharmaceutically relevant NPs can be highly streamlined. Similarly, such approaches could be applied to select the growth conditions that increase the presence of unknown molecular entities in crude-extract LC-MS analyses, thereby considerably increasing the chances of identifying unknown SMs.

An additional method which may prove helpful in helping to streamline cultivation based biodiscovery is microfluidics (Figure 1). Microfluidics employs methodologies and instrumentation to control and manipulate limited quantities of fluids in devices of a few centimeters in size, or microfluidic-chips, containing micrometer or even nanometer-wide channels and chambers [151,152]. In the past years, such approaches have found widespread application in areas ranging from genomics, medicine, chemistry, and immunology. The general principle behind these methods is to physically confine chemical or biological entities increasing the likelihood of controlling the processes under investigation. Both standard and custom made microfluidic-chips can be used, and the product of a specific chemical or biological process can be monitored using techniques such as mass spectrometry, fluorescence microscopy, and Raman micro-spectroscopy. For example, cells can be grown on a chip in micro-chambers, which are supplemented with a cultivation medium using an inlet channel (Figure 1). Outlet channels then connect the micro-chambers with microsolid phase extraction columns directly connected to high or ultra-high-resolution MS instruments [28]. In this way cellular metabolites can be directly extracted and analyzed using a single device, allowing a high level of parallelization. Even though these devices have not been directly developed with a biodiscovery purpose in mind, they are ideal for this purpose as they combine in a unique miniaturized solution all the steps required in the initial stages of the biodiscovery pipeline: cultivation of microorganisms, metabolite extraction, and metabolomic profiling via MS. While these technologies have not yet been explored as high-throughput approaches for the discovery of novel microbial NPs, we believe that they offer considerable potential, and promise a high degree of parallelization, low overall costs, and reduced timescales. In contrast, there are numerous examples of the use of microfluidic based approaches to test the bioactivity of chemical libraries [151,152]. When a target is identified (i.e., MRSA or prostatic cancer cells), microfluidic-chips can be used to simultaneously test the effect of multiple compounds on the target. The most common approaches use cell lines engineered with fluorescent proteins. In this way cellular proliferation within the microfluidic-chip can be detected as increased fluorescence by fluorescent microscopy. So far the two most common microfluidic approaches used micro-chambers or droplets. Droplets are formed within the microfluidic-chips as a consequence of the encounter of a water phase, usually containing cells and/or metabolites, and an oil carrier phase. Due to the immiscibility of these two phases, picolitre droplets are created. Both the droplets and micro-chambers are confined spaces in which both prokaryotic and eukaryotic cells can proliferate. When cells are confined in the presence of inhibitory molecules, their proliferation will be inhibited and the absence of increased fluorescence will indicate that the tested molecules have a toxic effect on the cell type being assayed [151]. For such applications, droplet-microfluidics offers even higher throughput than other types of microfluidics. In fact, thousands of droplets containing the target cells and the compound can be formed in a few minutes, subsequently collected; and re-analyzed following specific incubation times to detect proliferation (e.g. fluorescence). Subsequently, by exchanging the input chemicals and cell lines, which is as easy as changing the content of a syringe; new combinations can be tested offering unprecedented parallelization. During biodiscovery, bioassays can be highly time-consuming, this is especially true when high throughput cultivation such as micro-fermentations are employed, leading to many hundreds or thousands of crude extracts to be tested. Moreover, when the screening is not based on a specific target activity, multiple assays need to be undertaken; further increasing the experimental time required. The integration of micro-fermentation, micro-extraction, as used by Adnani et al. [27], and even robotic handling could greatly decrease the experimental time, parallelizing the work. In addition, due to its intrinsic high-throughput and the ease with which the input cells and extracts can be manipulated and exchanged, thus creating new ‘extract versus cell-line’ combinations, droplet-microfluidic represents an extremely promising technique for such purposes.

## 6. Concluding Remarks and Outlook

Zeeck and colleagues formalized in their “one strain many compounds” principle the strategies of fermentation optimization long since adopted in industrial microbiology [26]. Based on the same principle that a microbial strain is able to produce different natural products when cultivated under various conditions, additional strategies that do not simply modify the chemico-physical growth parameters, have been used. It is clear from the scientific literature that such approaches have not been extensively applied to marine microorganisms, which is surprising given that the marine environment and marine microorganisms in particular represent a promising source of novel NPs [5,7,141]. Among the approaches we have described, we feel that phosphate limitation, trace-element addition, co-cultivation and the addition of chemical elicitors represent the most promising strategies to help induce expression of genes encoding novel BGCs and the subsequent isolation and identification of novel NPs. Although limited to culturable microorganisms, these approaches take advantage of the cellular machinery of the organism hosting the “silent” BGCs. This allows for exploitation of the complex physiological interactions that can underpin SM production, accounting not only for the biosynthetic potential of a single BGC, but also for post-synthesis maturation processes, interaction between multiple BGCs, and interplay between primary and secondary metabolisms. Unless previously known, such phenomena are hard to replicate using pathways specific genetic engineering approaches. Moreover, these cultivation based approaches can also be easily applied to microbes that are not amenable to genetic manipulation, which is often a common feature among marine bacteria. It is clear that cultivation-based techniques suffer from the extensive experimentation needed to identify the right conditions triggering SM production, sometimes resulting in an overall long and costly route to undertake. However, mature technologies such as micro-fermentation and experimental design represent promising strategies that in combination will greatly help in streamlining the discovery processes. These methods can be integrated with genome-scale metabolic models, which allow the identification of parameters that are likely to affect the intensity of certain physiological processes (Figure 1). Genome-scale metabolic models aim to reconstruct the entire metabolic reactions and pathways of an organism. Stoichiometric factors and mass balances can be considered and this helps to predict which metabolic perturbation can result in a particular phenotype [153]. These methods have been increasingly applied in synthetic biology, but they have also helped in designing minimal growth media, and changing growth parameters to increase SM titrations [153,154]. For example, in actinobacteria, genome-scale metabolic reconstruction has helped to predict and validate the cultivation conditions needed to maximize production of the anti-diabetic compounds clavulanic acid and acarbose [153,154]. Similarly, the integration of other ‘omics’ data in the biodiscovery pipeline can help in gaining a better understanding of the regulatory mechanisms underpinning SM production, thereby helping with optimization of appropriate growth conditions. Growing “talented” microorganisms under various conditions and analyzing their transcriptome and proteome can help to verify which pathways are more affected by a specific set of changes, and can help to verify which “silent” BGCs are activated and thereby connecting SM production to expression of BGCs [143]. These technologies are now mature, leaving the cultivation throughput as the biggest bottleneck in cultivation-based approaches. The initial steps in the identification of the right media conditions to use in subsequent scale-up processes do not necessarily require high volumes. Therefore, miniaturization is undoubtedly the most promising strategy to achieve higher throughputs. Thus, microfluidic platforms may prove to be the best devices to help streamline the testing phases. The possibility to directly cultivating microorganisms in different media on the microfluidic-chip, combined with the possibility of performing micro-extraction and MS injection all on the same device, make such approaches highly attractive and demand that a more systematic effort be undertaken to embrace such cutting-age technologies within the biodiscovery pipelines. Overall it is an exciting time for NP discovery, and we believe that a more thorough integration of basic microbiological approaches, computational methods, and technological innovations will from a NP perspective, help to uncover much of the undoubted potential of marine microorganisms.
